# Load-Independent Characterization of Plate Foundation Support Using High-Resolution Distributed Fiber-Optic Sensing

**DOI:** 10.3390/s19163518

**Published:** 2019-08-11

**Authors:** Asmus Skar, Assaf Klar, Eyal Levenberg

**Affiliations:** 1 Department of Civil Engineering, Technichal University of Denmark, Nordvej, Building 119, 2800 Lyngby, Denmark; 2 Faculty of Civil and Environmental Engineering, Technion - Israel Institute of Technology, Haifa 32000, Israel

**Keywords:** distributed fiber-optic strain sensing, soil-structure interaction, foundation support, structural health monitoring, geotechnical analysis, pavement analysis

## Abstract

The evaluation of soil reaction in geotechnical foundation systems such as concrete pavements, mat- and raft foundations is a challenging task, as the process involves both the selection of a representative mechanical model (e.g., Winkler, Continuum, Pasternak, etc.) and identify its prevailing parameters. Moreover, the support characteristics may change with time and environmental situation. This paper presents a new method for the characterization of plate foundation support using high-resolution fiber-optic distributed strain sensing. The approach involves tracking the location of distinct points of zero and maximum strains, and relating the shift in their location to the changes in soil reaction. The approach may allow the determination of the most suited mechanical model of soil representation as well as model parameters. Routine monitoring using this approach may help to asses the degradation of the subsoil with time as part of structural health monitoring strategies. In this paper, fundamental expressions that relate between the location of distinct strain points and the variation of soil parameters were developed based on various analytical foundation support models. Finally, as an initial validation step and to underpin the idea basics, the proposed method was successfully demonstrated on a simple mechanical setup. It is shown that the approach allows for load-independent characterization of the soil response and, in that sense, it is superior to common identification methods.

## 1. Introduction

Common civil constructions, such as concrete pavements, mat- and raft foundations, involve precast or cast-in-place slabs resting on a prepared foundation support or so-called slab-on-grade construction. In engineering design, the mechanical behavior of slabs typically follows conventional elastic plate theory [1,2]. At the same time, the soil foundation behavior is most commonly represented by highly idealized response models, e.g., by employing the classical theories of elasticity and plasticity [3,4]. However, whereas the slab characteristics are engineered and well defined, the foundation support model is difficult to characterize.

In recent years, new sensing technologies have been developed for transforming conventional civil engineering structures into intelligent infrastructure. One leading technology in this connection is distributed fiber-optic strain sensing (see e.g., [5,6,7]). The development of this technology, and its capabilities to provide spatial profiles of strains along conventional telecommunication fibers, have led to a reevaluation of the manner in which strains can be used in civil engineering (see e.g., [8,9,10]).

Over the past decade, research in this area has been focusing on schemes of installation and interpretation of the spatially distributed data for various civil engineering problems, for example, pile foundations [11], evaluation of pipeline integrity to underneath excavation of a tunnel [12], stressing and deformation of secant pile walls [13], landslide localization [14], tunneling stressing [15], damage identification in concrete structures [16,17,18], strain measurement [19] and detection of cracks in asphalt pavements [20], and evaluation of tunneling induced ground deformations [21,22].

These interpretation methods are mostly based on analysis of static (slow-occurring) loading scenarios and on relatively low spatial resolutions of the order of one meter. However, new technological developments in this field now allows for a much higher spatial resolution at the order of a few millimeters [23,24,25,26,27,28,29,30]. This technological boost, not only enables new possibilities in structural health monitoring, but also makes the technology well suited for studying fundamental problems in a small-scale laboratory setting [31].

Foundation support models are relatively simple and easy-to-use approximations of the actual soil load-displacement response, and therefore play a significant role in geotechnical and pavement engineering research and practice (see e.g., [3,4,32,33,34,35,36]). It is generally assumed that, for serviceability design, the soil medium can be adequately represented by an elastic medium. For routine design purposes, Winkler’s idealization [37], characterized by a single parameter called coefficient of subgrade reaction *k*, has been used almost exclusively [38]. Another idealization assumes continuum behavior of the soil, and the soil medium is thus represented by an elastic half-space [39]. These two foundation support models can be regarded as the two extreme cases of soil behavior, represented on the one hand by the completely discontinuous medium (i.e., composed of discrete springs), and on the other by the completely continuous elastic solid. Thus, several simplified soil foundation models have been developed to provide a transition between these two types of idealized soil behavior [40,41,42,43,44]. This class of mathematical models has an additional constant parameter and, hence, the models are called two-parameter foundation models.

The effectiveness of using foundation support models for analysis of soil–structure interaction problems depends on the accuracy with which model parameters can be determined. Over the years, significant research effort has been devoted to the development of empirical expressions, linking the coefficient of subgrade reaction *k* to the properties of an elastic continua (see e.g., [45,46,47,48]), as well as realistic field conditions (see e.g., [49,50]). The modulus of elasticity is often determined from the early stages of triaxial load tests. Plate loading tests, and other non-destructive methods, may also be used to determine the in-situ modulus of elasticity of the soil. The elastic material properties can then be related in a theoretically rigorous fashion to the two-parameter foundation model parameters [38,51,52,53,54]. Methods for determining the parameters from non-standard field tests have also been proposed [41,42,55].

However, since all foundation support models are essentially idealizations, their fundamental assumptions may not be completely satisfied in actual field conditions. Moreover, taking into account the sensitivity of the support characteristics to temperature and moisture changes, the governing properties continually evolve under usual service conditions. Consequently, for any given plate foundation system, it is not straightforward to identify the governing support model, and the prevailing support properties at a given time and environmental situation.

Common means for foundation characterization are based on large-scale experiments [56,57]. These involve application of a load having known dimensions and intensity, and measurement of the resulting mechanical responses. Such procedure is, by very nature, expensive and service-disruptive; it is essentially relevant for sparse time intervals. As a means of addressing these limitations, the current work offers a new method for characterizing support conditions that is non-destructive, non-disruptive, and load-independent. The development has two purposes: (i) identify the support model type that best applies in a given situation, and (ii) characterize the associated foundation (soil) properties (i.e., quantify the coefficient of subgrade reaction and the intensity of shear interaction between the Winkler springs).

The suggested approach involves a loaded plate instrumented with distributed strain-sensing gear. It is based on the ability of tracking the location (relative to the loading location) of distinct points of zero and maximal bending moment. The relative locations of these points are essentially associated with the support parameters. In the paper, fundamental expressions that relate between the shift of strain of the distinct points and the variation of support model parameters are developed based on various static analytical plate solutions. Furthermore, the proposed methodology is experimentally validated by instrumenting a small-scale plate foundation system with high-resolution distributed fiber-optic strain sensors, capable of detecting the effects of loading events.

## 2. Plate Foundation Support Models

### 2.1. Modeling Idealized Soil Response

**Figure 1:**
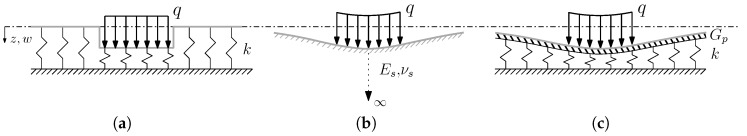
Response of foundation support models: (**a**) Winkler foundation model, consisting of independent springs characterized by a single parameter *k*, (**b**) the elastic half-space continuum model characterized by the Young’s modulus $E_{s}$ and Poisson’s ratio $\nu_{s}$, and (**c**) the Pasternak spring model with elastic layer capable of pure shear deformation, characterized by the two parameters *k* and $G_{p}$, respectively.

The complex behavior of a real soil mass has led to the development of many idealized models for soil behavior, especially for the analysis of soil–structure interaction problems. The Winkler model is simple and practical to many engineering problems and has therefore been used extensively for routine design of foundations and pavements [38]. In the Winkler model, the soil foundation properties are idealized as independent springs on a rigid base neglecting the effect of shear deformation between the springs, as shown in Figure 1a.

It is common experience that, in the case of soil media, surface deflections will occur not only immediately under the loaded region but also within certain limited zones outside the loaded region, as shown in Figure 1b. In attempts to account for this continuous behavior, soil media have often been idealized as three-dimensional continuous elastic isotropic solids. The first continuum representation of soil media stems from the work of Boussinesq [39].

However, both experimental and theoretical investigations have emphasized the need to provide a transition between these two types of idealized soil behavior since displacements outside the loaded region decrease more rapidly than that predicted by the elastic continuum model [3]. In this aspect, the mechanical two-parameter model proposed by Pasternak is attractive, shown in Figure 1c, offering an alternative to the elastic solid continuum by providing a degree of shear interaction between adjacent soil elements while remaining relatively simple to analyze [36].

### 2.2. Analytical Treatment of the Plate Foundation System

The mechanical behavior of slabs typically follows the classical Germain-Kirchhoff plate formulation [58]. Consider a plate of infinite size characterized by thickness *h*, Young’s modulus *E*, Poisson’s ratio ν, and therefore flexural rigidity *D*. The plate is loaded by a uniform vertical stress with intensity *q* distributed over a circular area with radius *a*. For this axisymmetric situation the vertical displacement field, $u_{z}$, depends only on the radial coordinate *r* with origin located directly under the load centroid. The plate moments (per unit length) in the radial ($M_{r}$) and tangential ($M_{\theta }$) are obtained from the expressions

(1) $$\begin{array}{cc} M_{r} & =-D\left(\frac{d^{2}u_{z}}{dr^{2}}+\frac{\nu }{r}\frac{du_{z}}{dr}\right)\\ M_{\theta } & =-D\left(\frac{1}{r}\frac{du_{z}}{dr}+\nu \frac{d^{2}u_{z}}{dr^{2}}\right) \end{array}$$

The corresponding radial and tangential bending stresses are $\sigma_{r}$ = $12zM_{r}/h^{3}$ and $\sigma_{\theta }$ = $12zM_{\theta }/h^{3}$, respectively, where *z* is measured from the plate’s mid-surface or neutral plane (positive = down). The extremal bending stresses are obtained at the bottom of the plate where *z* = h/2 and at the surface where *z* = −h/2. The notation is such that a positive moment is associated with tensile bending stress at the plate bottom under the loaded area, i.e., where *r* = 0 and z=h/2. Finally, radial strain ($\varepsilon_{r}$) and the tangential strain ($\varepsilon_{\theta }$) at a given *z* are obtained from

(2) $$\begin{array}{cc} \varepsilon_{r} & =\frac{12z(M_{r}-\nu M_{\theta })}{Eh^{3}}\\ \varepsilon_{\theta } & =\frac{12z(M_{\theta }-\nu M_{r})}{Eh^{3}} \end{array}$$

In the case of a Pasternak foundation type, the vertical displacement field is [59]

(3) $$u_{z}=\frac{qa}{lk}\int_{0}^{\infty }\frac{J_{0}\left(mr/l\right)J_{1}\left(ma/l\right)}{m^{4}+2bm^{2}+1}dm$$

where $l=\sqrt[{4}]{D/k}$ is the so-called radius of relative stiffness [60]. *k* is the coefficient of subgrade reaction (force/length^3^), $J_{n}\left(\;\right)$ denotes a Bessel function of the first kind of order *n*, *m* is a unitless integration variable or wave number, and *b* = $G_{p}/2kl^{2}$ is positive and dimensionless wherein $G_{p}$ (force/length) is the second parameter, and represents the intensity of the shear interaction between the Winkler springs. For the special case of *b* = 0, or equivalently $G_{p}$ = 0, the expression provides the solution for a plate on Winkler foundation. The corresponding plate bending moments (per unit length) in the radial and tangential directions are

(4) $$\begin{array}{cc} M_{r} & =\frac{qaD}{l^{2}kr}\int_{0}^{\infty }\frac{mJ_{1}\left(ma/l\right)\left(\left(mr/l\right)J_{0}\left(mr/l\right)-\left(1-\nu \right)J_{1}\left(mr/l\right)\right)}{m^{4}+2bm^{2}+1}dm\\ M_{\theta } & =\frac{qaD}{l^{2}kr}\int_{0}^{\infty }\frac{mJ_{1}\left(ma/l\right)\left(\left(\nu mr/l\right)J_{0}\left(mr/l\right)+\left(1-\nu \right)J_{1}\left(mr/l\right)\right)}{m^{4}+2bm^{2}+1}dm \end{array}$$

Hogg assumed a plate with frictionless bottom bonded to a linear elastic isotropic half-space [61], herafter referred to as the half-space continuum model. The vertical displacement field is [3]

(5) $$u_{z}=\frac{2qa(1-\nu_{0}^{2})}{l_{e}E_{s}r}\int_{0}^{\infty }\frac{J_{0}\left(mr/l\right)J_{1}\left(ma/l\right)}{m^{4}+m}dm$$

where $E_{s}$ and $\nu_{s}$ are the elastic properties of the half-space support, and $l_{e}=\sqrt[{3}]{2D\left(1-\nu_{s}^{2}\right)/E_{s}}$ is the characteristic length associated with the a plate on a half-space (analogous to the radius of relative stiffness). The corresponding plate bending moments (per unit length) in the radial and tangential directions are

(6) $$\begin{array}{cc} M_{r} & =\frac{2qa(1-\nu_{0}^{2})}{l_{e}E_{s}r}\int_{0}^{\infty }\frac{mJ_{1}\left(ma/l\right)\left(\left(mr/l\right)J_{0}\left(mr/l\right)-\left(1-\nu \right)J_{1}\left(mr/l\right)\right)}{m^{3}+1}dm\\ M_{\theta } & =\frac{2qa(1-\nu_{0}^{2})}{l_{e}E_{s}r}\int_{0}^{\infty }\frac{mJ_{1}\left(ma/l\right)\left(\left(\nu mr/l\right)J_{0}\left(mr/l\right)+\left(1-\nu \right)J_{1}\left(mr/l\right)\right)}{m^{3}+1}dm \end{array}$$

## 3. Proposed Interpretation Method

This section describes a method for characterising the foundation parameters based on distributed fiber-optic strain measurements of the radial strain ($\varepsilon_{r}$) at the top of plate (*z* = −h/2). It is assumed that the strains can be detected by fiber-optic cables attached to the slab. The methodology is composed of three elements: (i) a mechanical plate foundation model, (ii) distributed fiber-optic strain measurements, and (iii) an iterative interpretation scheme.

**Figure 2:**
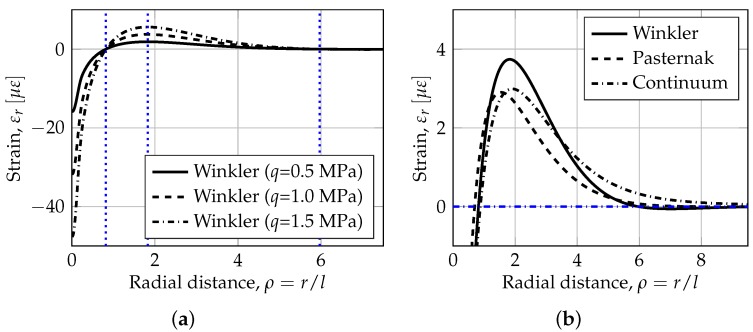
Influence of foundation model type on radial strain response for a standard concrete support plate system: (**a**) overview of strain response as a function of radial distance and (**b**) close-up of the region around first and second zero crossing.

To exemplify the overall strain response of a standard support plate system, the radial strain is plotted versus the normalized radial distance (ρ) from the loaded point, shown in Figure 2a for three different load intensities (i.e., *q* = 0.5–1.5 MPa). In the Figure it was assumed that slabs are constructed on a 150 mm thick high-quality sub-base over subgrade soil. Moreover, the slab is composed of concrete having a Young’s modulus *E* = 30,000 MPa and a Poisson’s ratio ν = 0.15. It’s thickness is *h* = 300 mm, leading to a flexural rigidity of *D* = 6.91 × 10^10^ Nmm. Figure 2b shows the effect of the model on the the radial strain at the top when the slab is loaded by a single heavy wheel with radius, *a* = 150 mm. The foundation support parameters are given as: (i) Winkler-model: *k* = 0.055 MPa/mm, (ii) Pasternak-model: *k* = 0.055 MPa/mm and *b* = 0.5, and (iii) half-space continuum model: $E_{s}$ = 102 MPa and $\nu_{s}$ = 0.35. Some points of interest are also included with dotted blue lines, these are hereafter referred to as Distinct Points (DPs) of zero strain DP${}_{0,i}^{j}$ (i.e., $\rho \left(\varepsilon_{0,i}\right)$), and maximum strain DP${}_{m,i}^{j}$ (i.e., $\rho \left(\varepsilon_{m,i}\right)$), where *i* = 1, 2 is the number of zero/maximum strain location from the center of the load, and *j* = ‘W’, ‘P’, ‘C’, indicating the support type Winkler, Pasternak and Continuum, respectively. Zero strain is shown as a dashed dotted blue line along the abscissa-axis. The radial coordinate is normalized by the radius of relative stiffness (*l*) and characteristic length ($l_{e}$) both equal to 1059 mm.

From Figure 2a it is observed that the loading intensity *q* has no effect on the results. Moreover, separate analysis has shown that the results are essentially insensitive to the exact radius of loading as long as it is of the same order as the plate thickness or smaller (down to a point load). It was also found that the results are insensitive to the value of $\nu_{s}$.

In Figure 2b a close-up of the strain response in the region around the first and second zero crossing is shown. A few characteristic features are revealed in the figure w.r.t. the distinct points for the different model types, i.e.: (i) the Winkler-model has a first (DP${}_{0,1}^{W}$) and second zero crossing (DP${}_{0,2}^{W}$) at a radial distance from the load of ≈*l* and 6l, respectively, (ii) the Pasternak-model has a first crossing at a radial distance lower than the Winkler-model (i.e., DP${}_{0,1}^{P}$ < DP${}_{0,1}^{W}$), whereas the second crossing is larger (i.e., DP${}_{0,2}^{P}$ > DP${}_{0,2}^{W}$), and (iii) the half-space continuum model as a first zero crossing larger than the Winkler-model (i.e., DP${}_{0,1}^{C}$ > DP${}_{0,1}^{W}$) and no second zero crossing. It is also found that the magnitude of the second peak for the Winkler-model is higher than for the Pasternak-model and the half-space continuum model (i.e., $\varepsilon_{m,2}^{W}>\varepsilon_{m,2}^{P,C}$) due to the lack of shear transfer in the supporting medium.

**Figure 3:**
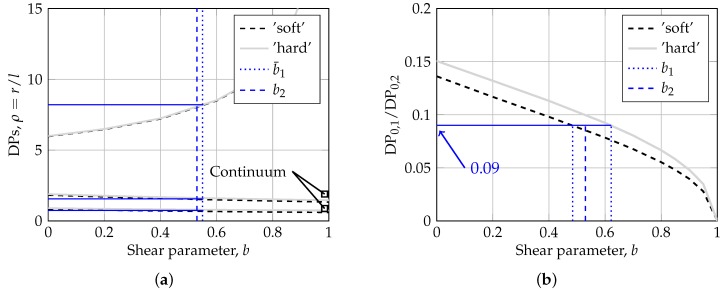
Parametric study of the Pasternak-model: (**a**) Influence of Pasternak’s *b* parameter on the normalized locations of the DPs defined in Figure 2 for two cases (**b**) influence of Pasternak’s *b* parameter on the location ratio of first to second zero crossings defined in in Figure 2 for the two cases.

The observations above show that the location of distinct points are closely related to the governing foundation model type and to the numerical values of the model parameters. To further investigate these features, the Pasternak-model is utilized as proposed in [62], considering two different cases, spanning two extreme yet realistic situations. The first case considered is a very thick concrete plate resting on a very ‘soft’ spring-bed with a large radius of relative stiffness of *l* = 2000 mm. The second case considered is a very thin concrete plate resting on a very ‘hard’ spring-bed support with a short radius of relative stiffness of *l* = 336 mm. First, the location of the distinct points are plotted versus the shear interaction parameter *b*, shown in Figure 3a. Presented next is the location ratio of the first to the second zero crossings (i.e., DP${}_{0,1}$/DP${}_{0,2}$) as a function of *b* for the two cases, shown in Figure 3b.

From Figure 3a it can be seen that the results for the two cases essentially overlap, i.e., that the location of distinct points are relatively insensitive to the value of *l*. The first zero crossing location is influenced by *b*, dropping from about 0.86*l* at *b* = 0 to about 0.67*l* at *b* = 1. The second peak location also drops with increasing *b*, 1.86*l* at *b* = 0 to about 1.40*l* at *b* = 1. A pronounced dependence on *b* is exhibited by the location of the second zero crossing, increasing from about 5.89*l* at *b* = 0 towards infinity as *b* approaches unity. It is also found that as *b* increase, the discrepancy between the Pasternak-model and the half-space continuum model increases. This shows that although the Pasternak-model possess some of the characteristic features of continuous elastic solids, it is a simplification which cannot capture all complexities. Finally, from Figure 3b it is observed that the two curves do not collapse onto one unique line, the gap between them is considered small, establishing an almost unique relationship between the ratio and parameter *b*.

The relations shown in Figure 3 form the basis of the non-disruptive fiber-optic based test method for characterizing the plate support conditions suggested in herein. Specifically, the problem is to determine *k* and *b* such that a best match is achieved between measured and modeled location of distinct points/strain response.

The starting point for the method is identifying the locations of the distinct points (see Figure 2). This step is non-destructive, non-disruptive, load-independent (given that the load magnitude and radius are not needed), and can be done repeatedly. Next, Figure 3b is entered for the first iteration, with the location ratio of the first to second zero crossings to provide a range of estimated values for the shear parameter, i.e., $b_{1}$, marked with blue dotted lines. The average of this range (i.e., ${\bar{b}}_{1}$) is then utilized in Figure 3a to resolve the value of the radius of relative stiffness *l*. Once *l* is identified, a second iteration is to be carried out to refine the estimation of the shear parameter (and subsequently *l*), i.e., $b_{2}$, marked with a blue dashed line in the Figure. Finally, given that the plate properties are known, it becomes possible to calculate the Winkler coefficient of subgrade reaction $k=Dl^{-4}$, and the intensity of shear interaction between the Winkler springs $G_{p}=2kbl^{2}$.

In the Figure the procedure proposed are shown for the plate foundation system in Figure 2 with k=0.055 MPa/mm and *b* = 0.5 (i.e., the ‘Pasternak-model’). The distinct points are calculated as DP${}_{0,1}$ = 736.5 mm, DP${}_{m,2}$ = 1637.2 mm and DP${}_{0,1}$ = 8204.6 mm which yields a location ratio of the first to the second zero crossings of 0.09, and resulting foundation model parameters *k* = 0.064 MPa/mm, *b* = 0.54 and *l* = 1017. Thus, *k*, *b* and *l* was estimated with 17%, 8% and 4% accuracy, respectively.

## 4. Experimental Investigation

### 4.1. Experimental Setup

In order to validate the proposed interpretation method a small-scale experiment was designed applying high-resolution distributed fiber-optic strain sensing. The test was designed and carried out to provide a first-order demonstration of the proposed characterization concept with off-the-shelf equipment; it was not designed to mimic real-life situation. Therefore, practical aspects such as embedding fiber optic cables in concrete and dealing with multiple load situations were not considered.

**Figure 4:**
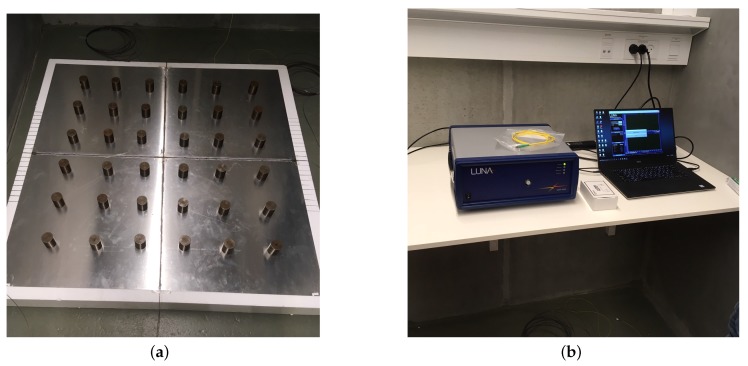
Small-scale slab on grade system: (**a**) aluminum plate instrumented with fiber-optic cables and supported by a thick polystyrene mat and (**b**) OBR device and laptop.

The experimental setup consisted of an aluminum plate over a finite thickness support material on a concrete floor, shown in Figure 4a. The aluminum plate was instrumented with a fiber-optic cable, glued to the top of the plate in both directions (at right angles), and connected to the measurement device on one end. Finally, a dead-load was applied, using 36 × 0.5 kg weights placed in a grid, to ensure contact between the plate and the support material. Since the loading and theory are axisymmetric, the fibre lines in the experiment were positioned to capture radial strains and not in a grid arrangement. A grid arrangement is envisioned for field applications, where the load position cannot be a priori known. The availability of a strain grid can be utilized to identify the loading location based on a criterion of maximal bending strain. Afterwards, strain analysis is to be performed according to the proposed theory for fibres that measure radial strains.

The plate was loaded at the center (i.e., far from the edges) with hand-held weights on a small rubber pad with area, $A_{\mathrm{load}}$ = 36 mm^2^. The load was applied at the center of the test plate to minimize edge effects and better comply with the theoretical derivation. Strain measurements were then recorded with a commercial Optical Backscatter Reflectometer (OBR) device [63] depicted in Figure 4b. Strain values were recorded in intervals of 1mm with a gauge length of 10 mm. In the specific case, the load is of short time duration compared to changes in the support, and thus, no temperature compensation is needed. In cases where loads are stationary for a long period of time, e.g., in the case of foundations, temperature compensation will be needed.

A thin rubber mat and a thick polystyrene mat were selected as foundation material in an attempt to resemble the two outer extreme cases, i.e., a Winkler-type and a Continuum-type foundation, hereafter referred to as support type ‘thin’ and ‘thick’, respectively. Moreover, a thin flexible plate, was selected to limit the size of the test setup, and at the same time, comply with the plate formulation presented in Section 2 (i.e., avoid edge effects). The geometrical and material properties for the different structural elements of the system are given in Table 1.

### 4.2. Experimental Results

**Figure 5:**
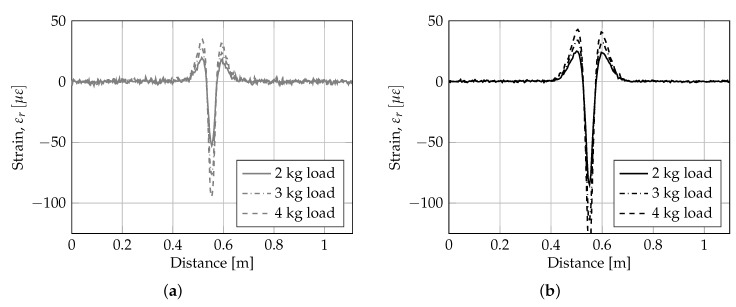
1-D representation of raw fiber-optic strain measurements at different load levels (2–4 kg) for (**a**) support type ‘thin’ (gray) and (**b**) support type ‘thick’ (black).

The raw fiber-optic strain measurements are presented in the 1-D plots in Figure 5. In the Figure the strain data for support type ‘thin’ (gray lines) and ‘thick’ (black lines) are plotted at a load level of 2–4 kg along one of the lengths of the aluminum plate.

Comparing the raw strain signal for foundation support type ‘thin’ and ‘thick’ in Figure 5a,b, respectively, it is observed that the overall shape is similar and symmetric. However, the noise level is higher for foundation type ‘thin’, and also higher than the expected/specified level of app. ±1 με. Thus, subsequent analysis of data were performed on strain measurements averaged over 4 load and unloading tests (i.e., for noise reduction). Moreover, for further analysis a load of 4 kg (i.e., q≈ 1.09 MPa) was selected in order to maximize the signal to noise ratio.

**Figure 6:**
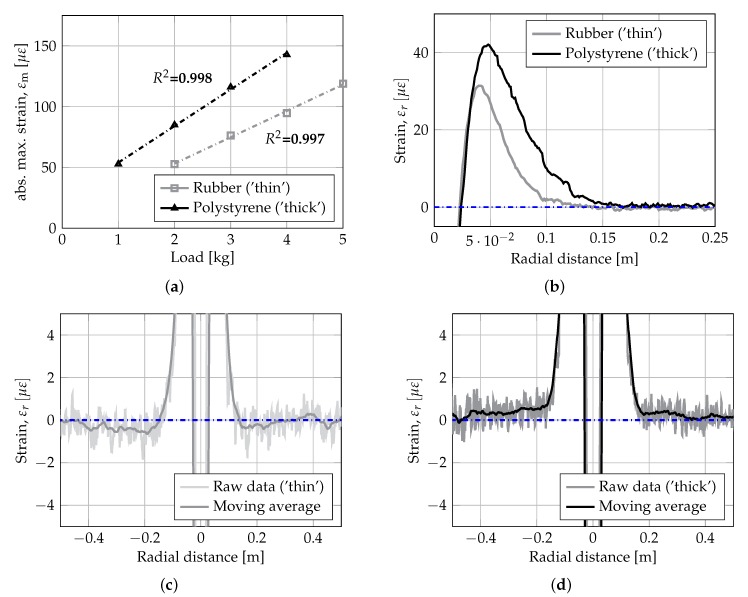
Comparison of distributed fiber-optic strain measurements for different support materials: (**a**) linearity of materials, (**b**) close-up of region around first and second zero crossing, (**c**,**d**) close-up of second zero crossing, showing point and moving average strain measurements for foundation type ‘thin’ and ‘thick’, respectively.

In Figure 6, the detailed experimental results are presented. Figure 6a shows the peak strains measured for the two support types at four different load levels. Figure 6b shows the effect of the support type on the developed strain. Finally, Figure 6c,d present a close-up the raw data signal and the moving average of strain measurements is shown for support type ‘thin’ and ‘thick’, respectively. The moving average data is calculated using a base length of $L_{b}$ = 50 mm.

From Figure 5a, it is observed that both plate foundation systems behave linearly for the applied loading magnitudes. This is a basic prerequisite for further analysis of the strain data using the proposed framework, avoiding influence of shift in distinct points due to nonlinear behavior. In Figure 2b a close-up of the strain response in the area around the first and second zero crossing is shown. From the Figure it is observed that the support type ‘thick’ has a first zero crossing slightly larger than support type ‘thin’, i.e., DP${}_{0,1}^{‘\mathrm{thick}’}$ = 24.4 mm > DP${}_{0,1}^{‘\mathrm{thin}’}$ = 19.9 mm. Moreover, support type ‘thin’ has a clear zero crossing at DP${}_{0,2}^{‘\mathrm{thin}’}$ = 130.9 mm, whereas support type ‘thick’ has a no clear zero crossing, although the abscissa is crossed at a radial offset of app. ±400 mm. Thus, the two support types ‘thin’ and ‘thick’ show some of the characteristic features of the Winkler-model and the half-space continuum model, respectively (i.e., equivalent to a Pasternak-model with high and low shear interaction). The location of the second peak is DP${}_{m,2}^{‘\mathrm{thin}’}$ = 37.9 mm and DP${}_{m,2}^{‘\mathrm{thick}’}$ = 47.0 mm.

### 4.3. Interpretation of Results

The results obtained from the small-scale experiment are next interpreted using the iterative scheme proposed in Section 3. First, the results visualised in Figure 3 are reproduced for the aluminium plate with a flexural rigidity of *D* = 2.16 × 10^4^ Nmm and load radius *a* = 3.38 mm. The support conditions are taken as *k* = 0.001 MPa/mm and *k* = 10.0 MPa/mm to ensure a sufficient upper and lower limit of *l*, i.e., 68.10 mm and 6.81 mm, respectively. The analytical results for the experimental plate support system, i.e., the location of the distinct points, as well as the ratio of first zero crossing to second zero crossing, are shown in Table 2.

The ratio of first zero crossing to second zero crossing is 0.152 and 0.063, for support type ‘thin’ and ‘thick’, respectively. These are entered in Table 2, and the corresponding parameters $b_{1}^{‘thin^{\prime }}$ = 0–0.019 and $b_{1}^{‘thick^{\prime }}$ = 0.751–0.845 are found by interpolation (marked with gray cells in the Table). These values are then used to provide a first estimate of the shear parameter of ${\bar{b}}_{1}^{‘\mathrm{thin}’}$ = 0.010 and ${\bar{b}}_{1}^{‘\mathrm{thick}’}$ = 0.798 after averaging, providing three different estimates of the distinct points (marked with gray cells in the Table 2). Consequently, the radius of relative stiffness is estimated, via averaging of the three different possible values, to be 27.2 mm and 33.82 mm, for support type ‘thin’ and ‘thick’, respectively.

Next, another iteration is performed with 0.152 and 0.063, and a value of $b_{2}^{‘\mathrm{thin}’}$ = 0.005 and $b_{2}^{‘\mathrm{thick}’}$ = 0.843 is obtained via interpolation (considering that $l_{1}^{‘\mathrm{thin}’}$ = 21.72 mm and $l_{1}^{‘\mathrm{thick}’}$ = 33.82 mm). The location of the distinct points are reentered, this time with $b_{2}^{‘\mathrm{thin}’}$ = 0.005 and $b_{2}^{‘\mathrm{thick}’}$ = 0.843, and the above described calculations are repeated. The final result is $l_{2}^{‘\mathrm{thin}’}$ = 21.71 mm and $l_{2}^{‘\mathrm{thick}’}$ = 32.77 mm, which leads to a modulus of subgrade reaction of $k^{‘\mathrm{thin}’}$ = 0.097 MPa/mm and $k^{‘\mathrm{thick}’}$ = 0.019 MPa/mm.

The results of the analysis are summarized in Table 3. Specified material properties (see Table 1) and measured values are shown in brackets. The expression for the characteristic length (see Section 2) is utilized for calculating the ‘equivalent’ Young’s modulus for each support type.

From Table 3, it is observed that predicted Young’s modulus for support type ‘thin’ and ‘thick’ is 3.36 MPa and 1.22 MPa, respectively, matching well the known material properties (see Table 1). Moreover, the estimated shear parameter *b* is 0.005 and 0.843, respectively, indicating that the ‘thin’ support system is dominated by compression (i.e., the support material acts similar to the Winkler-model), whereas the ‘thick’ support system is highly affected by shear. Thus, the methodology enables identification of suitable model type. It is also found that the estimated location of distinct points match well with the experimental values. This outcome provides basic confidence in the proposed method and experimental results obtained.

**Figure 7:**
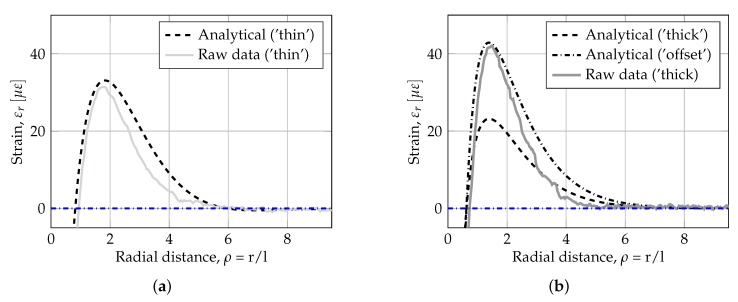
Comparison of analytical results with estimated support conditions and experimental fiber-optic strain measurements: (**a**) Pasternak-model with *k* = 0.097 MPa/mm and *b* = 0.005 vs. rubber foundation (‘thin) and (**b**) Pasternak-model with *k* = 0.019 MPa/mm and *b* = 0.843 vs. polystyrene foundation (‘thick). In generating these plots the loading intensity of *q* = 1.09 MPa was used.

To visualize the results the analytical (using the estimated model parameters from Table 3) and experimental strain curves are plotted in the region around the first and second zero crossings, shown in Figure 7.

From Figure 7a, it is observed that the analytical strain response resembles the experimental curve. The discrepancy between curves is especially pronounced after the second peak. This could indicate that other effects (not only compression and shear) also influence the system. One potential effect is friction, as the coefficient of friction in the experiment (i.e., between rubber and aluminium) is much higher than actual field conditions (i.e., between sand subbase/polyethylene sheet and concrete). The potential effect of friction was investigated in a separate analysis utilizing a detailed Finite Element (FE) model of the problem. The FE computations showed that friction between the aluminium plate and foundation material have little influence in the location of the first zero crossing. However, increasing friction results in an increasing negative slope after the second peak. The shift was 0–10%, decreasing rapidly as ρ→∞. Thus, increasing friction has a positive effect on the overall fit between analytical and experimental results.

From Figure 7b, it is observed that the magnitude of the second peak (i.e., $\varepsilon_{m,2}$) is significantly lower than the experimental peak strain. This discrepancy can be explained by the sensitivity of the experimental setup to offsets in the position of the fiber-optic cable relative to the plate’s mid-surface (i.e., position of strain measurement), e.g., created by small variations in thickness of the film of gluing between fiber-optic cable and plate. The difference in peak strain is equivalent to an offset error of app. 0.4 mm, exemplified in the Figure with a dashed dotted curve named ‘offset’. Thus, this error is negligible for real world applications. Furthermore, it is observed that the difference in the position of strain measured do not influence the position of distinct points, showing the robustness of the methodology selected.

## 5. Conclusions

This study focused on the development of a non-destructive interpretation method for characterizing the plate foundation support conditions using static analytical foundation support models and high-resolution distributed fiber-optic strain sensing.

The proposed methodology was based on tracking a few distinct points of zero and maximum strain. This is the first time such a tracking idea has been utilized for parameter identification in geotechnical infrastructure. This approach has the advantage that it allows for load-independent characterization of the soil response, and in that sense, it is superior to other system identification methods that rely on response magnitudes.

As a first step towards routine engineering application, the method was tested and validated in a small scale experiment of aluminium plate resting on a thin rubber and thick polystyrene support systems. The experimental results showed that a second zero crossing was identified for the thin rubber foundation. Whereas, the strain approaches zero after the second peak (as the radial distance increases), and that no clear second zero crossing can be identified for the polystyrene foundation. These findings comply well with the theory that the thin rubber should resemble a Winkler support system while the thick polystyrene a continuum domain.

In the experiments presented, the third peak for foundation type ‘thin’ was ≈1 με, whereas the theoretical third peak in realistic concrete plate on foundation systems is ≈0.1 με. In this aspect identification of the second zero crossing may be difficult considering a real-scale plate foundation health monitoring system. On the other hand, true sized problems do not require such high-resolution, and a resolution of a few centimeters should be sufficient to clearly identify the characteristic points. It can also be shown that the highly idealized modeling result in discrepancy in the overall strain response in the region around the second peak. This is a result of the materials selected for the conceptual small-scale experiment presented in this paper (i.e., high friction coefficient between materials). In realistic slab-on-grade construction friction contact will likely have a minor effect (i.e., considering the relatively low friction between soil and structure).

The current research demonstrated, both theoretically and experimentally, that shape features of the spatial strain profile (under load) contain relevant information for foundation characterization. Thus, a conceptually novel monitoring technique can be envisioned that is non-destructive, non-disruptive, and load-independent.

There are many challenges in line before the idea can be applied in real life situations, e.g., sensing placement, sensing resolution, and sensing range. The present work serves as an initial first step towards a full-scale health monitoring, underpinning the idea basics, and therefore identifying those practical aspects that require further development. The next development phase should involve application of the proposed interpretation method to real soils and validation against an independent measurement system. Future efforts should also be expended on improving the technology, enabling analysis of moving and dynamic loads, as well as fiber-optic optimization for finding the required trade-off between resolution and accuracy. The methodology provided within this paper can be the basis for such future research efforts.

## Figures and Tables

**Table 1:** Geometrical and material properties used in experimental study.

Structural Element	Material	Young’s Modulus	Poisson’s Ratio	Thickness	Length	Width
		[MPa]	[-]	[mm]	[mm]	[mm]
Plate	Aluminium	68,300	0.33	1.5	1100	1100
Support (‘thin’)	Rubber [64]	2–4	0.45	10.0	1250	1250
Support (‘thick’)	Polystyrene [65]	1–2	0.05	100.0	1200	1200

**Table 2:** Location of the distinct points for the aluminium plate support system.

	l = 68.10 mm					l = 6.81 mm				
b	DP${}_{0,1}$	DP${}_{m,2}$	DP${}_{0,2}$	DP${}_{0,1}$/DP${}_{0,2}$	b	DP${}_{0,1}$	DP${}_{m,2}$	DP${}_{0,2}$	DP${}_{0,1}$/DP${}_{0,2}$	
0				0.152						
0	0.811	1.821	5.962	0.136	0	0.924	1.910	6.006	0.154	
0.01	0.808	1.815	5.986		0.01	0.921	1.904	6.030	→	$l_{1}^{‘thin^{\prime }}$ = 21.72 mm
					0.02				0.152	
0.20	0.753	1.694	6.456	0.117	0.20	0.879	1.798	6.504	0.135	
0.40	0.705	1.585	7.202	0.098	0.40	0.843	1.703	7.254	0.116	
0.60	0.664	1.490	8.477	0.078	0.60	0.813	1.622	8.532	0.095	
0.70	0.645	1.447	9.561	0.067	0.70	0.800	1.585	9.617	0.083	
0.75				0.063						
0.80	0.628	1.408	11.350		0.80	0.788	1.552	11.408	→	$l_{1}^{‘thick^{\prime }}$ = 33.82 mm
0.80	0.628	1.407	11.384	0.055	0.80	0.788	1.551	11.442	0.090	
					0.85				0.063	
0.85	0.620	1.387	12.941	0.048	0.85	0.783	1.535	12.982	0.060	
0.90	0.613	1.369	15.242	0.040	0.90	0.777	1.520	15.591	0.050	
0.95	0.605	1.351	21.533	0.029	0.95	0.772	1.505	21.730	0.037	
1.00	0.598	1.333	*∞*	0.000	1.00	0.767	1.491	*∞*	0.000	

**Table 3:** Summary of analysis results.

Support Type	DP${}_{0,1}$	DP${}_{m,2}$	DP${}_{0,2}$	*k*	*b*	$E_{s}$	$\nu_{s}$
	[mm]	[mm]	[mm]	[MPa/mm]	[-]	[MPa]	[-]
‘thin’	18.8 (19.9)	40.4 (37.9)	130.2 (130.9)	0.097	0.005	3.38 (2–4)	(0.45)
‘thick’	23.0 (24.4)	48.0 (47.0)	417.8 (400.0)	0.019	0.843	1.22 (1–2)	(0.05)
